# Single-Cell RNA-Seq Reveals the Promoting Role of Ferroptosis Tendency During Lung Adenocarcinoma EMT Progression

**DOI:** 10.3389/fcell.2021.822315

**Published:** 2022-01-20

**Authors:** Jiaxi Yao, Yuchong Zhang, Mengling Li, Zuyu Sun, Tao Liu, Mingfang Zhao, Zhi Li

**Affiliations:** ^1^ Department of Medical Oncology, The First Hospital of China Medical University, Shenyang, China; ^2^ Department of Urology, The First Hospital of China Medical University, Shenyang, China; ^3^ Department of Clinical Epidemiology and Center of Evidence-Based Medicine, The First Hospital of China Medical University, Shenyang, China

**Keywords:** lung adenocarcinoma, single-cell sequencing, ferroptosis, epithelial-mesenchymal transition, causal inference

## Abstract

Epithelial-mesenchymal transition (EMT) and ferroptosis are two important processes in biology. In tumor cells, they are intimately linked. We used single-cell RNA sequencing to investigate the regulatory connection between EMT and ferroptosis tendency in LUAD epithelial cells. We used Seurat to construct the expression matrix using the GEO dataset GSE131907 and extract epithelial cells. We found a positive correlation between the trends of EMT and ferroptosis tendency. Then we used SCENIC to analyze differentially activated transcription factors and constructed a molecular regulatory directed network by causal inference. Some ferroptosis markers (GPX4, SCP2, CAV1) were found to have strong regulatory effects on EMT. Cell communication networks were constructed by iTALK and implied that Ferro_High_EMT_High cells have a higher expression of SDC1, SDC4, and activation of LGALS9-HARVCR2 pathways. By deconvolution of bulk sequencing, the results of CIBERSORTx showed that the co-occurrence of ferroptosis tendency and EMT may lead to tumor metastasis and non-response to immunotherapy. Our findings showed there is a strong correlation between ferroptosis tendency and EMT. Ferroptosis may have a promotive effect on EMT. High propensities of ferroptosis and EMT may lead to poor prognosis and non-response to immunotherapy.

## Introduction

Lung cancer is the most commonly diagnosed cancer and the leading cause of cancer death, which includes small cell lung cancer (SCLC) and non-small cell lung cancer (NSCLC). Lung adenocarcinoma (LUAD) is the most prevalent NSCLC. Currently, the main treatment for advanced LUAD including targeted therapy, radiotherapy, chemotherapy and immunotherapy. Despite the application of new therapies in recent years, advanced LUAD remains prone to metastasis, resulting in a poor prognosis with a 5-year survival rate of less than 20%. Therefore, further research on the mechanisms of lung cancer metastasis is urgently needed.

One of the most familiar factors in the metastasis of tumors is epithelial-mesenchymal transition (EMT). During EMT, epithelial cells lose their original polarity and intercellular adhesion and acquire invasive properties associated with the mesenchyme (Chen et al., 2021). Furthermore, it can contribute to tumor progression and has been recognized as a driver of tumor spread in many types of tumors (Puram et al., 2017). Current research suggests that EMT transcription factors like SNAI1, TWIST1 and ZEB1 may contribute to the promotion of tumor metastasis and drug resistance by EMT (Chen et al., 2021). EMT is associated with resistance to chemotherapy and immunotherapy, it is therefore considered to play an important role in the treatment of tumors (Panchy et al., 2019; Bulle and Lim, 2020). It has been previously published that in NSCLC, EMT may lead to exhaustion of CD8^+^ T cells, resulting in immunosuppression (Soundararajan et al., 2019). EMT also interacts with a variety of biological processes, leading to metastasis and poor prognosis. As a result, extensive research has been carried out on the relationship between EMT and other biological processes in recent years.

Ferroptosis is a newly identified iron-dependent form of programmed cell death caused by unrestricted lipid peroxidation and plasma membrane rupture (Dixon et al., 2012). The accumulation of iron, production of free radical and toxic lipid peroxides are the main features of ferroptosis, which was verified to be related to a variety of molecular mechanisms (Wu et al., 2021a). Cells underwent ferroptosis may bring an abundance of oxidized lipids, which can affect the phagocytosis of dendritic cells and the presentation of antigens, and then cause immune evasion of tumor cells (Demuynck et al., 2021). Previous research has shown EMT can increase cellular sensitivity to ferroptosis. On the one hand, it has been known that a selective vulnerability to ferroptosis is related to highly mesenchymal-like cell state (Viswanathan et al., 2017). Protein LYRIC can promote EMT, while at the same time, promote ferroptosis by inhibiting GPX4 (Bi et al., 2019). On the other hand, ferroptosis tendency also contributes to EMT (Du et al., 2021). Erastin, which is an inducer of ferroptosis, can act on lung epithelial cells and causes de-epithelialize (Sun et al., 2021). PTGS2 is another ferroptosis promotor, the silencing of which can inhibit the proliferation and migration of pancreatic cancer cells (Xu et al., 2021a). Combined with the conclusions in the previous literatures, EMT and ferroptosis tendency may form a positive feedback loop within a certain range. However, the existing studies were conducted based on cell line experiments that lack of complex tumor microenvironment, which may lead to results that are too artificial to address clinical questions. *In vivo* environment, the relationship between ferroptosis tendency and EMT has been little studied and is not yet clear. Hence, there is an urgent need for a more precise analysis.

Single-cell RNA sequencing (scRNA-seq) can partly solve the problems above. scRNA-seq uses samples obtained from human body for analysis and can represent the results of *in-vivo* experiments. It is a high-resolution tool that overcomes the limitations of traditional bulk sequencing, allowing us to measure tumor heterogeneity at the level of individual cell and is valuable for the study of the tumor microenvironment (Xu et al., 2021b; Liu et al., 2021). Previous studies of vitro experiments have shown that ferroptosis is involved in the EMT process, but the exact regulatory mechanism is unclear *in vivo*. Therefore, we used scRNA-seq to assess the relationship between ferroptosis tendency and EMT in LUAD.

In data analysis after scRNA-seq, we get many correlation conclusions. Various statistical models, machine learning, and deep learning models are used to get various correlations through analysis, but correlation cannot represent the objectivity and authenticity of causality. Correlation indicates two variables are related, which show an increase or decrease trend (Altman and Krzywinski, 2015). Causality is where the cause is partly responsible for the effect, and the effect is partly dependent on the cause. The main difference between causal inference and correlation inference is that the former analyzes the response of the effect variable when the cause is changed (Pearl, 2009; Vemuri, 2015). In addition, at present, regulation is only verified at single molecular level, probably because for basic experiments, the overall regulatory network is difficult to construct. To clarify the dependence of different biological processes, causal inference is increasingly used in biological data such as genome, proteome, and scRNA-seq data to explain the regulatory network between molecules (Sella et al., 2017).

Here, we used scRNA-seq data analysis and found that ferroptosis tendency in tumor cells is closely related to EMT. At the same time, we used SCENIC and causal inference to construct a directed network for key TFs and markers, and found that the tendency of ferroptosis has a positive regulatory effect on EMT, which plays an important role in tumor proliferation, metastasis and ineffective immunotherapy. This result will provide important support for the clinical treatment decision of LUAD.

## Results

### EMT and Ferroptosis in mLN are Positively Correlated

**FIGURE 1:**
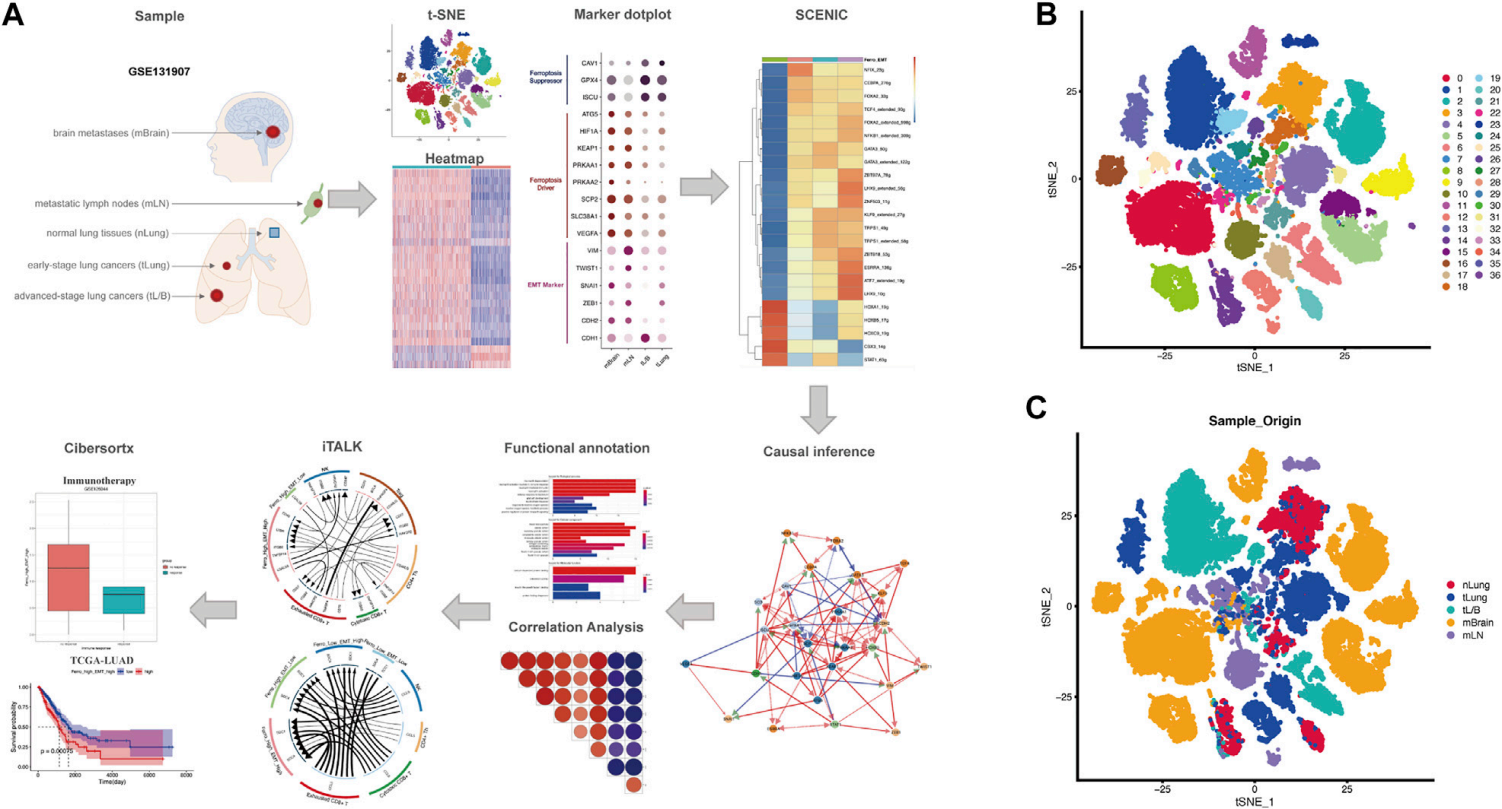
Overall flowchart and t-SNE dimensionality reduction clustering. **(A)** Flowchart of the work. **(B,C)** t-SNE plots showed the clustering results and the different origins of epithelial cells, including nLung, tLung, tL/B, mBrain, mLN.

Processed scRNA-seq data were screened with strict QC criteria in the origin article, and we use the original meta. data file to annotate all cells (Kim et al., 2020). 36,071 ECs from different multiple histologic sections were obtained for our follow-up analysis (Figure 1A). After performing gene filtering, normalization, principle component analysis, we first set the resolution at 0.7, resulting in the 37 cell clusters, and visualized the original samples utilizing t-SNE plots (Figures 1B,C).

**FIGURE 2:**
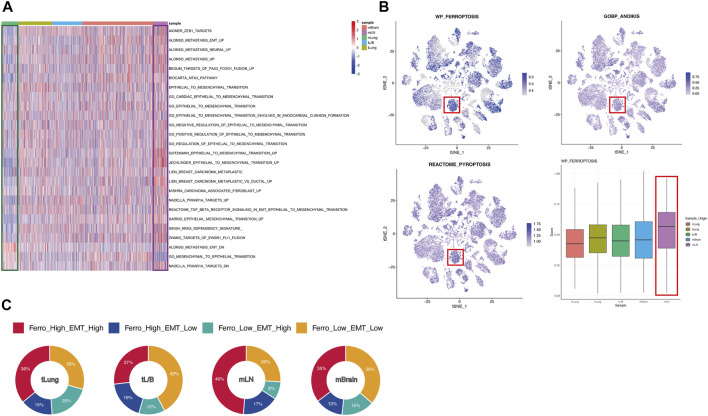
EMT and ferroptosis were significantly active in mLN. **(A)** Heatmap showed the expression of EMT-related genes in cells of different origins. ssGSEA score of EMT was low in normal tissues (green box) and high in mLN (purple box). **(B)** t-SNE plot showed the score of various forms of cellular death. Box plot showed ferroptosis tendency scores of cells from different tissue sources. mLN had the highest score (red box). **(C)** All ECs was divided into four groups based on the median score: Ferro_High_EMT_High, Ferro_High_EMT_Low, Ferro_Low_EMT_High, Ferro_Low_EMT_Low and are summarized in the pie chart.

EMT is a cell reprogramming process, which ECs acquire a mesenchymal phenotype. A series of changes activated after EMT help tumor cells to invade surrounding tissues and metastasize (Dongre and Weinberg, 2019; Pastushenko and Blanpain, 2019). Since the tumor tissues come from different metastatic sites, we put the research center on EMT, using 23 EMT up-regulated and 3 EMT down-regulated gene sets obtained from MSigDB to perform ssGSEA on all ECs (Subramanian et al., 2005). The analysis revealed that the expression of EMT-related gene set was down-regulated in normal tissues (green box), while globally up-regulated in tumor derived tissues, especially in mLN (purple box) (Figure 2A). This result suggests that there are some EMT-generating cells in the tumor tissue, which may cause the primary tumor metastasis of lung cancer. Considering EMT was strongly associated with ferroptosis tendency, we further explored various forms of cellular death including ferroptosis using the ssGSEA method (Figure 2B). Compared with other forms of death, we found that ferroptosis has specific activation in different tissues. In mLN, the ferroptosis pathway in most ECs is activated, which was the same as the activation of the EMT pathway (Figure 2B). It is worth noting that some cells in mBrain and other sample tissues are also highly sensitive to ferroptosis. However, unlike mLN, in other tissues, only a part of cells exhibited a high sensitivity to ferroptosis. This may have some relation to the tumor microenvironment. Additionally, the heterogeneity in other tissues such as mBrain may also be a reason for this inconsistency. To further confirm the relationship between ferroptosis tendency and EMT, we divided all ECs into four groups based on the median score: Ferro_High_EMT_High, Ferro_High_EMT_Low, Ferro_Low_EMT_High, Ferro_Low_EMT_Low (Figure 2C). Through the pie chart, we can clearly see that the proportion of “Ferro_High_EMT_High” cells (double-high group) in mLN was as high as 49%, which suggested that there was a strong correlation between EMT and ferroptosis in mLN.

### Marker Expression and Enrichment Analysis Suggested a Strong Correlation Between Ferroptosis Tendency and EMT in all Epithelial Cells

**FIGURE 3:**
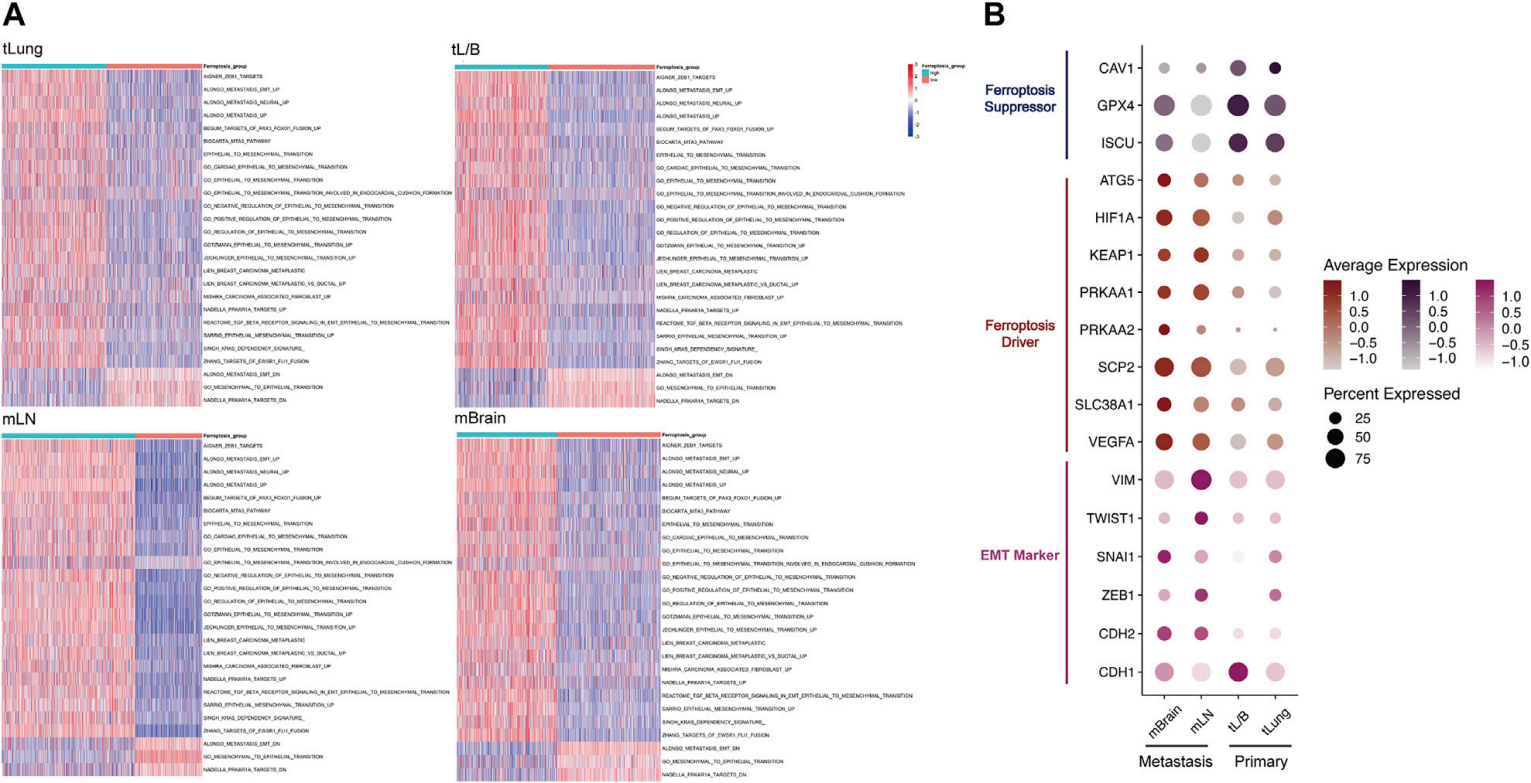
In tLung, tL/B, mLN, mBrain, EMT all exhibited homotropic changes with ferroptosis. **(A)** Heatmap showed the EMT score was significantly increased in the group with high ferroptosis tendency score group. **(B)** Dotplot also indicated a strong association between the expression of EMT markers and ferroptosis markers in four original sites. Darker dots suggest higher average expressions.

Among the proportions of cells from the four original samples, we found that the sum of the proportions of the double-high group and the double-low group accounted for about 70% (Figure 2C). This result proved that ferroptosis and EMT might occur simultaneously in ECs. In order to prove that this result was not unique in mLN, we divided all ECs into “high” and “low” groups according to the median ssGSEA score of ferroptosis, and all ECs were grouped and analyzed according to original samples (Figure 3A). We found that the EMT score was significantly increased in the group with high ferroptosis group, no matter in the primary tumor (tLung, tL/B) or the metastatic site (mLN, mBrain). This result proves that EMT displays homotropic cooperativity with ferroptosis tendency are not only in metastatic lymph nodes, but are closely related in all lung cancer cells, which is a common phenomenon.

Next, we detected the markers of EMT and ferroptosis in the four original samples (Figure 3B; Supplementary Table S1). The ferroptosis suppressor (CAV1, GPX4, ISCU, ATG5) and ferroptosis driver (HIF1A, KEAP1, PRKAA1, PRKAA2, SCP2, SLC38A1, VEGFA) were obtained through FerrDB database (http://www.zhounan.org/ferrdb/) (Zhou and Bao, 2020). EMT markers (downregulated: CDH1; upregulated: CDH2, ZEB1, SANI1, TWIST1, VIM) were selected according to previous literature (Thiery et al., 2009; Zhang et al., 2021a). We used dotplot to display the above markers expressions in different original samples (Figure 3B). We found a strong correlation between the expression of EMT markers and ferroptosis markers in metastasis and primary tumors. Compared with the primary tumor, in the metastasis group, the mesenchymal markers (CDH2, VIM, TWIST1, SNAI1, ZEB1) and ferroptosis drivers increased, and the epithelial marker CDH1 and ferroptosis suppressor decreased. This result once again confirmed from the level of molecular markers that the relationship between EMT and ferroptosis tendency played a vital role in tumor metastasis process.

### Causal Inference Network Suggested Ferroptosis Tendency Promoted the Occurrence of EMT

**FIGURE 4:**
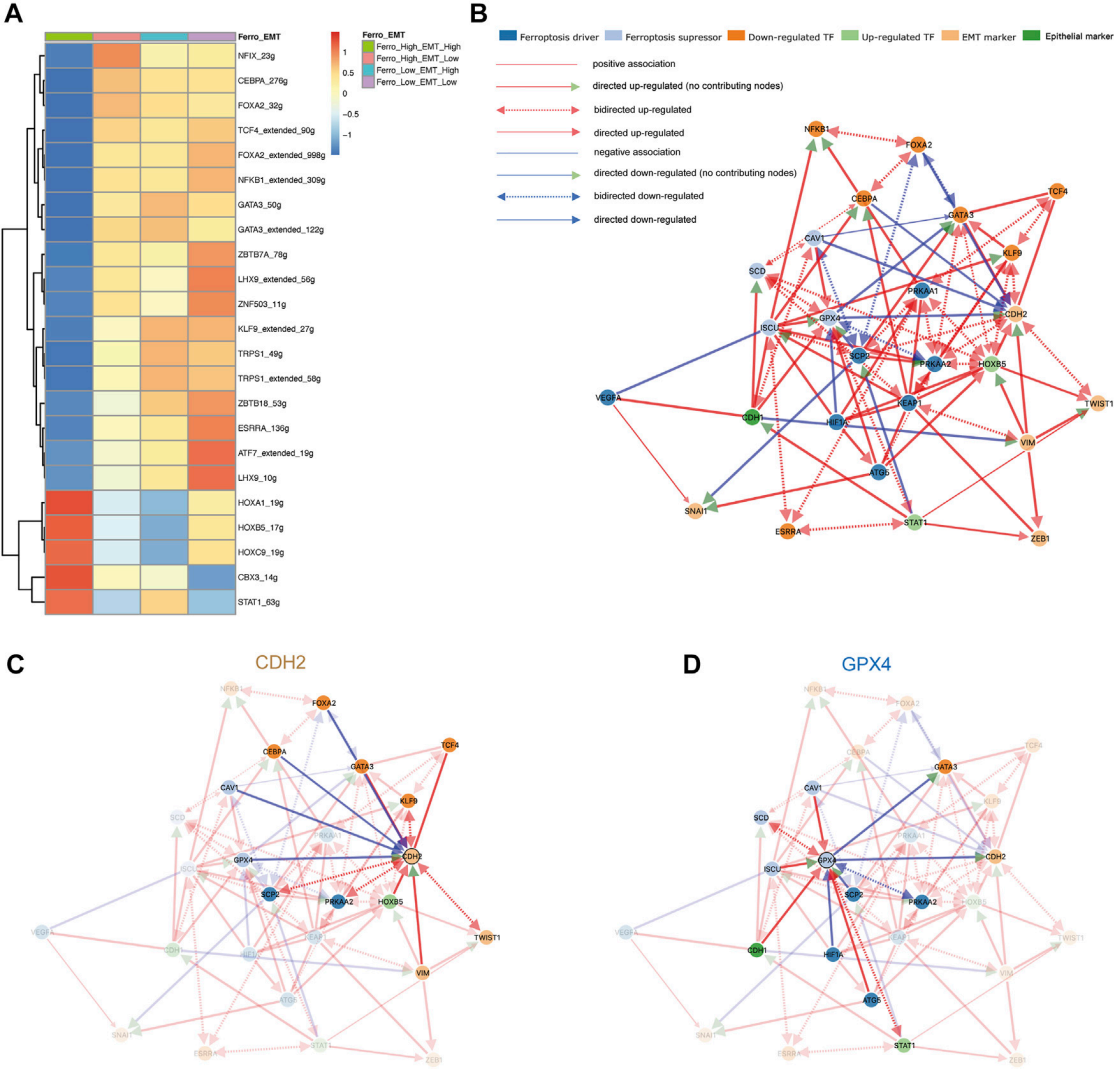
Ferroptosis tendency may promote the occurrence of EMT. **(A)** SCENIC analysis revealed transcription factors regulatory information in the four groups. 5 up-regulated (red) and 18 down-regulated (blue) transcription factors were detected in the double-high group. **(B)** The cause-effect relationships among transcription factors, ferroptosis and EMT markers. **(C,D)** The causal inference network shows regulatory relationships demonstrated around CDH2 and GPX4. GPX4 can negatively regulate the expression of CDH2, while CDH1 can up-regulate the expression of GPX4.

In order to explore the transcriptional regulatory network inside the double-high group, we then used SCENIC to infer transcription factors (TFs) regulatory information underlying each EC phenotype. SCENIC analysis revealed that some TFs had obvious differential activation and deactivation in the double-high group. We then compared the double-high group with other groups, and detected 5 up-regulated and 18 downregulated TFs (Figure 4A). In the double-high group, we can clearly see that the HOX family genes (HOXA1, HOXB5, HOXC9) is highly expressed. HOX family genes regulate the proliferation, migration and invasion of tumor cells by regulating the transcription of target genes, and participate in the occurrence and development of tumors and drug resistance (Zhang et al., 2003; Bhatlekar et al., 2014; Zhang et al., 2018). In addition, CBX3 is also closely related to metastasis and poor prognosis (Zhong et al., 2019). Further, decreased expression of down-regulated TFs, such as (GATA3, TRPS1, NFKB1, FOXA2, KLF9, etc.), also indicates the influence of the double-high group on poor prognosis and metastasis (Tang et al., 2011; Chou et al., 2013; Gong et al., 2018; Low et al., 2020; Wang et al., 2021).

In order to further analyze the regulatory relationship among transcription factors, ferroptosis and EMT markers, we first constructed an undirected network graph through String (version 11.5) and visualized using Cytoscape (version 3.8.2) software (Supplementary Figure S1A). By deleting disconnected nodes in the network, we used connected genes as variables for causal inference (Supplementary Table S2). Figure 4B provided an overview of directed network graph to describe the cause-effect relationships between selected genes. It was apparent from Figures 4C,D that GPX4 as an ferroptosis suppressor could negatively regulate the expression of CDH2, which means that promoting ferroptosis tendency will promote the EMT of malignant epithelial cells. In addition, CDH1 as an epithelial marker can also up-regulate the expression of GPX4 and promote the tendency of ferroptosis. Some other regulatory relationships (SCP2, HOXB5, CAV1) also confirm that ferroptosis can positively regulate the occurrence of EMT (Supplementary Figures S1B–S1D). According to previous literature, during the EMT process, the high transcription level of SNAI1, TWIST1 and ZEB1 will increase the sensitivity of cells to ferroptosis (Chen et al., 2021). Taken together, we believe that EMT of epithelial cells will increase the sensitivity of ferroptosis, and the occurrence of ferroptosis can enhance the transformation of epithelial cells to mesenchymal cells, which form a positive feedback mechanism loop (Xu et al., 2021a; Sun et al., 2021). To validate the clinical relevance of molecules in the gene regulatory network, we performed a validation of the ferroptosis marker in the TCGA-LUAD database. GPX4, as a suppressor of ferroptosis, has an HR of 0.74, *p* < 0.05. While PRKAA2 as a driver of ferroptosis has an HR of 1.37, *p* < 0.05 (Supplementary Figures S1E,F). In other words, some markers of ferroptosis can lead to poor prognosis in lung adenocarcinoma, which confirms that the promotion of EMT by ferroptosis leads to poor prognosis.

### A Certain Level of the Intracellular Reactive Oxygen Species (ROS) is Generated in Double-High Group

**FIGURE 5:**
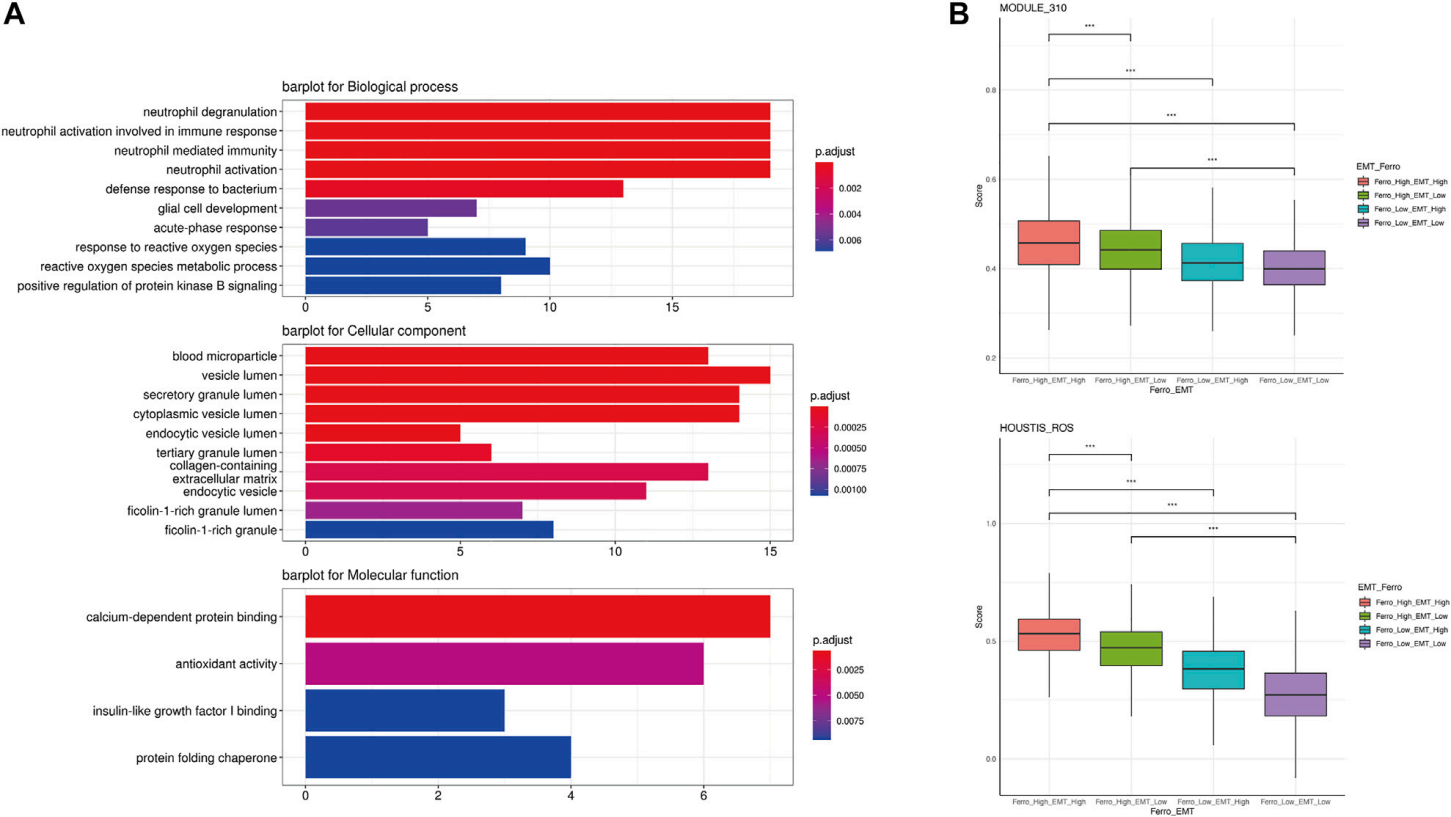
A higher accumulation of ROS occurred in the double-high group. **(A)** GO enrichment analysis showed some activated ROS-related biology progresses and molecular functions (“neutrophil degranulation,” “response to reactive oxygen species,” “calium-dependent protein binding,” “antioxidant activity”). **(B)** ssGSEA indicated higher enrichment scores of two ROS-related genesets in the double-high group.

In order to further analyze the differences between the double-high group and other groups, we first screened DEGs through the Findmarker function in Seurat (Supplementary Table S3). Next, we used ClusterProfiler 4.0 for GO enrichment analysis (Figure 5A). The enrichment analysis results showed that some ROS-related biology progresses and molecular functions were activated (“neutrophil degranulation,” “response to reactive oxygen species,” “calium-dependent protein binding,” “antioxidant activity,” etc.) (Dou et al., 2015; Clemens et al., 2017; Sakurada et al., 2019; Yang et al., 2019). We used ssGSEA to score the enrichment of two ROS-related genesets (“HOUSTIS_ROS,” “MODULE_310”), which also shown that the accumulation of ROS in the double-high group was higher (Figure 5B).

Several reports have shown that ROS cause changes in downstream molecular such as Src, FAK and 15-PGDH, leading to the activation of EMT in multiple tumors (Wang et al., 2018; Cheung et al., 2020; Peng et al., 2020). Interestingly, the process of ferroptosis was often accompanied by an increase in ROS level and a decrease in the antioxidant system (Zheng and Conrad, 2020; Yao et al., 2021a). As the site for intracellular oxidative respiration, mitochondrion is a major source of ROS. By promoting mitochondrial respiration, the ferroptosis promoter erastin can increase the production of ROS and thus promote ferroptosis by enhancing mitochondrial respiration (Yao et al., 2021b). Combined with the results of the above causal inference, we believe that ferroptosis leads to the low expression of GPX4, which promotes the accumulation of ROS and reduces the inhibition of CDH2 expression, thereby promoting the occurrence of EMT. According to previous literature, we believe that there is a critical value for this promoting. When the strength of promoting ferroptosis is higher than that of EMT, malignant epithelial cells are more likely to undergo programmed death *in situ* instead of EMT.

### Cells Communications Revealed Proliferative Activity and Non-Response to Immunotherapy of Double-High Group

**FIGURE 6:**
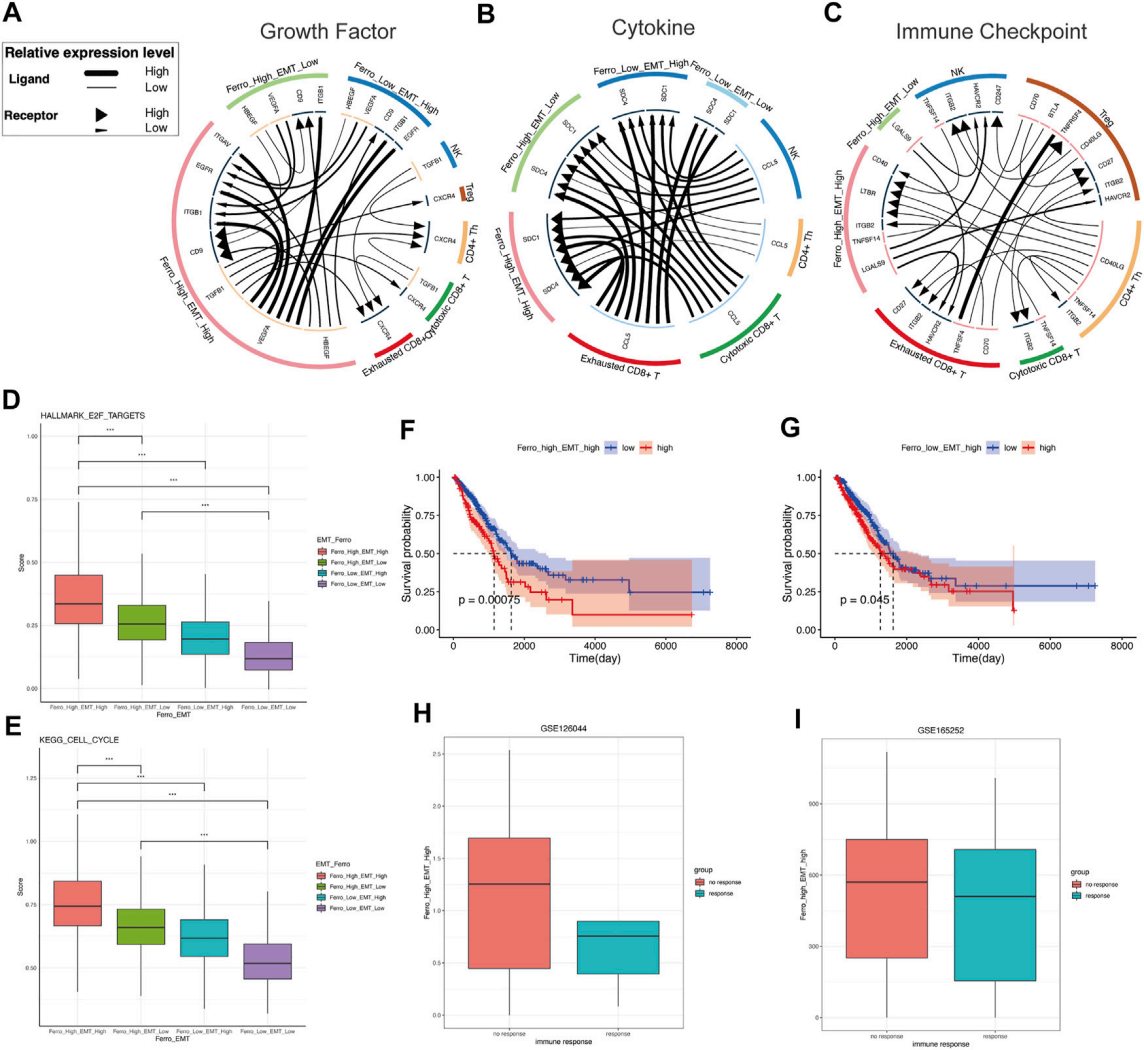
The double-high group showed higher proliferative activity and non-response to immunotherapy. **(A)** Intracellular and intercellular communications is more active in the double-high group in growth factor module of cell communication networks. The expression of VEGFA, HBEGF, TGFB1, CD9 is higher than other groups. **(B)** Cytokine module of cell communication networks showed higher expressions of SDC1 and SDC4 in the double-high group. **(C)** In immune checkpoint module of cell communication networks, the interaction of LGALS9-HARVCR2 between tumor cells and immune cells is more activated in the double-high group. **(D,E)** The cell cycle and E2F targets scores of the double-high group were also significantly higher than those of the other groups. **(F,G)** Survival curves showed that the double-high group was linked to a poorer overall survival, and is even worse than the “Ferro_Low_EMT_High” group. **(H,I)** Both two LUAD immunotherapy cohorts (GSE126044, GSE165252) showed that the non-response group has more double-high group cells than the response group.

To describe the molecular association between epithelial cells and lymphocytes, we first constructed cell communication networks based on the interaction of potential receptor-ligand pairs using iTALK. Depending on the function of receptor-ligand pairs, we evaluate cellular interactions from three modules: “Growth Factor,” “Cytokine,” and “Immune Checkpoint” (Figures 6A–C).

In growth factor module, it is clear that there are significant intracellular and intercellular communications in the double-high group (Figure 6A). This result suggests the stimulating effect of autocrine or paracrine growth factors on double-high group cells. Compared to other groups, the double-high group has more VEGFA, HBEGF, TGFB1, CD9 expression. We found that double-high group cells potentially stimulated tumor growth and metastasis through angiogenesis signaling molecules (VEGFA-ITGB1, VEGFA-EGFR, VEGFA-ITGAV) (Maae et al., 2012; Chou et al., 2013; Kim et al., 2017; Sullivan et al., 2019). A specific activation of TGFB1-CXCR4 of the double-high group revealed a high degree of EMT (Xu et al., 2009). The high expression of CD9 also suggests that cancer cells will be more aggressive and rapidly form large tumors (Wang et al., 2019a). Because of the activation of multiple growth factor pathways, we further analyzed the cell cycle (Figures 6D,E). We found that the proliferation of the double-high group also increased significantly. In cytokine module, we found that SDC1 and SDC4 had higher expressions in the double-high group than in other groups (Figure 6B). The high expression of SDC1 and SDC4 indicates proliferation, metastasis and drug resistance of tumor cells, which will result in a poor prognosis (Yao et al., 2019; Kim et al., 2021). Pearson correlation was applied to analyse the correlation of ssGSEA sore of the malignant epithelial cells (Supplementary Figure S2A).

In the immune checkpoint module (Figure 6C), we found that an increase in ferroptosis tendency score would lead to a specific interaction of LGALS9-HARVCR2 between tumor cells and immune cells, with more activation in the double-high group than in the single-high (Sun et al., 2020; Zhang et al., 2021b). A large number of studies have shown that the activation of LGALS9-HARVCR2 in various tumor tissues is related to the development of tumors and negatively regulation of immune signaling. The activation of the LGALS9-HAVCR2 signal can co-express with PD-1 in tumor-infiltrating immune cells, and cooperate to mediate effector T cell exhaustion and dysfunction (Bi et al., 2021; Dixon et al., 2021). This result suggests that double-high group cells may cause non-response to immunotherapy.

### Evaluate the Impact of Double-High Group on Survival and Immunotherapy Efficacy

To investigate the clinical role of the cell classification identified in this study, we used CIBERSORTx to evaluate the scores of each cell type in the TCGA LUAD cohort (Supplementary Table S4). “Ferro_High_EMT_Low” group suggested a good prognosis, while the “Ferro_Low_EMT_Low” group (double-low group) had no significant difference in OS (Supplementary Figure S2B). High EMT was related to poor overall survival (OS) (Figures 6F,G). However, it was striking that the cells in the double-high group resulted in worse OS than the “Ferro_Low_EMT_High” group. It was worth noting that the double-high group also had a higher enrichment score in patients with metastases (Supplementary Figures S2C,S2D). The above results once again confirmed that ferroptosis may have a positive regulation on EMT.

Cell communication results indicated that there was a close relationship between immune cell and double-high group cells. Therefore, we chose LUAD immunotherapy cohorts (GSE126044, GSE165252) to verify the therapeutic effect. Consistent with the results of cell communication, more double-high group cells were seen in the immunotherapy non-response group (Figures 6H,I). Our results predicted that the double-high group cells promote tumor metastasis and lead to a poor prognosis, and cause non-response to immunotherapy.

## Discussion

Ferroptosis and EMT are closely related as two important biological processes, but the relationship between the two needs to be further confirmed. Therefore, this article used scRNA-seq technology to analyze the correlation between EMT and ferroptosis tendency from the single-cell level. Then, we used causal inference network to objectively interpret the promotion effect of ferroptosis activation on EMT in more details.

Some literature has proved EMT-related signaling contributes to ferroptosis *in vitro* experiments. ZEB1 is one of the major transcription factors that regulate EMT by binding to the E-box in E-cadherin (Larsen et al., 2016). In the mesenchymal state of cells, ZEB1 correlates with the sensitivity of GPX4 inhibition and thus increases the susceptibility to ferroptosis (Chen et al., 2021). On the contrary, E-cadherin can mediate cell-to-cell contact and inhibit EMT while protecting cells from ferroptosis (Wu et al., 2019). Susceptibility to ferroptosis can be restored when expression of EMT-promoting transcription factors such as SNAI1, TWIST1 and ZEB1 is elevated. On the contrary, ferroptosis tendency can enhance the development of EMT. When PTSG2, as a driver of ferroptosis, was blocked, the expression of N-cadherin was suppressed and the expression of E-cadherin was promoted, which suggested that inhibition of ferroptosis can lead to inhibition of EMT in parallel (Xu et al., 2021a). Not coincidentally, the ferroptosis promoter Erastin reduced the expression of E-cadherin, again suggesting that ferroptosis tendency may be engaged in the development of EMT (Sun et al., 2021). However, the above results were obtained from cell line experiments and were not validated *in vivo* experiments. Our study used single-cell sequencing technology, with samples from human tissues, which is closer to the clinical patient’s condition and has a greater reference value for clinical treatment. At the same time, we confirmed that ferroptosis tendency has a strong effect on EMT through more objective and true causal inferences. We used difference analysis to identify DEGs between the double-high cells and other cells, and enrichment analysis suggested that ROS played an important role in the mutual promoting of EMT and ferroptosis (Figures 5A,B).

In the study of transcriptional regulation, we used the technique of SCENIC and causal inference. We presented GPX4 plays a negative regulatory role on CDH2. There are also some other meaningful regulatory relationships in the transcriptional regulatory network, which also reveals that ferroptosis tendency can promote EMT (Supplementary Figure S1B). SCP2, as a ferroptosis driver, was found to promote other ferroptosis drivers (PRKAA1, PRKAA2) and EMT marker (CDH2), while inhibiting ferroptosis suppressors such as GPX4 and CAV1. Transcription factors play a key role in the regulation of biological behavior, and HOXB5 is one of them. In lung cancer, it was observed *in vitro* experiment that proliferation and metastasis of NSCLC cell lines were significantly inhibited when HOXB5 was knocked down (Zhang et al., 2018). Similar results can be found in HNSCC. It has been suggested that HOXB5 regulates EGFR transcription by binding to the EGFR promoter region and therefore it could act as an oncogenic driver in HNSCC (Lee et al., 2020). We found that HOXB5 also promoted the activation of EMT and ferroptosis in the regulatory network (Supplementary Figure S1C). However, there are currently few reports of a link between ferroptosis and HOXB5, and we believe that this may be a direction that can be explored in the future. CAV1 promotes the expression of GPX4 and CDH1, and inhibits CDH2, which implies that it suppresses ferroptosis and EMT (Supplementary Figure S1D). Previous studies have also reported that decrease of CAV1 can promote EMT, more strongly validating the reliability of our results (Zhang et al., 2016). We also verified the correlation between each molecule in bulk sequencing, which is consistent with our results (Supplementary Figure S3).

Immunotherapy has been an important treatment for lung cancer in recent years, but patients’ response to it has been variable and the reasons are not sufficiently clear. By deconvolution of single cell data, we found an immunosuppressive effect of double-high group cells in immunotherapy. In our study, we have seen that LGALS9-HARVCR2 was significantly expressed in both the double-high group and the Ferro_High_EMT_Low group, suggesting that elevation of ferroptosis may play a role in the suppression of immunotherapy. We can also observe that the expression of LGALS9 was higher in the double-high group than in Ferro_High_EMT_Low group, demonstrating a stronger inhibition of immunotherapy in the double-high group. This may provide some clues to the poor efficacy of immunotherapy in some patients and can be a reliable predictor of the risk of immunotherapy. Some of these internal molecular modulations may provide a good direction for future clinical decisions on immunotherapy. Another phenomenon associated with the immune microenvironment is that in tumorigenesis, ferroptosis plays a dual role in promoting and inhibiting tumors, which is attributed to the release of damage-associated molecular patterns (DAMPs). Immunogenic cell death occurs as a result of the release of DAMPs, thereby stimulating anti-tumor immunity (Galluzzi et al., 2017). At the same time, however, the inflammatory response is promoted, thus supporting the growth of tumors (Tang et al., 2012; Chen et al., 2021).

Notably, the current single-cell methods for constructing gene regulatory networks (GRN) using causal inference are time-series-based expression and scRNA-seq expression. MIIC is a scRNA-seq expression profile-based causal inference method, which is based on mutual information for causal inference. This method is essentially based on curve fitting and may have statistical error with the real results. Although the EMT marker SNAI1 received inhibition from SCP2, which is different from the finding that ferroptosis have a promotive effect on EMT, we consider this situation as an uncertain association that does not affect the final results. MIIC, as a scRNA-seq expression profile-based method for constructing gene regulatory networks, can achieve a high accuracy and still shows better results in our GRN.

In summary, we demonstrated the contribution of ferroptosis tendency to EMT through a variety of advanced technologies, and we found that the mutual promotion between them may lead to tumor metastasis, growth and non-response to immunotherapy.

## Materials and Methods

### Samples and Epithelial Cells Extraction

The data used for this research were downloaded from online public database, which present no ethical issues. We obtained processed single-cell LUAD data from GEO data sets (GSE131907) (Kim et al., 2020). The clinical information of the patients (Supplementary Table S5) and metadata (Supplementary Table S6) come from the original study.

To evaluate EMT enrich score of each epithelial cell, we extracted all epithelial cells (ECs) for subsequent analysis. Because the number of ECs derived from PE is small, we extract normal lung tissues (nLung), early-stage lung cancers (tLung), advanved-stage lung cancers (tL/B), metastatic lymph nodes (mLN) and brain metastases (mBrain) for subsequent analysis (Figure 1A).

### Single Cell RNA-Seq Data Quality Control and Data Processing

According to the original articles, malignant tumor cells were separated from non-malignant cells in tumor tissues and three quality measures were applied on raw matrix for each cell: mitochondrial genes (≤20%, unique molecular identifiers (UMIs), and gene count (ranging from 100 to 150,000 and 200 to 10,000). A total of 2000 highly variable genes identified by the FindVariableFeatures function in the Seurat (version 4.0.3) package were used for principal component analysis (PCA)-based dimensionality reduction with RunPCA (Hao et al., 2021). t-distributed stochastic neighbor embedding (t-SNE) was utilized to visualize single-cell clustering using principal components (PCs) 1 to 30. Dotplot function was used to display the expression of EMT and ferroptosis markers in different samples.

### Transcription Factor Regulatory Analysis and PPI Network

Single-cell regulatory network inference and clustering (SCENIC) is a new computational method to map genes regulatory networks and identify stable cell states by evaluating the activity of each cell from scRNA-seq data (Aibar et al., 2017). SCENIC was performed on 2000 cells sampled from all ECs, and the regulons were calculated based on transcription factors (TFs) or their target genes. Only regulons significantly upregulated or downregulated were involved in further analysis.

We constructed a protein-protein interaction network for a total of 37 genes (5 upregulated and 14 downregulated TFs, 12 ferroptosis markers, 6 EMT markers) using String version 11.5 (https://string-db.org) (Szklarczyk et al., 2019), and visualized with the force-directed layout algorithm by Cytoscape software (version 3.8.2) (Shannon et al., 2003).

### Causal Inference Network

Multivariate Information-based Inductive Causation (MIIC) algorithm relies on a novel information-theoretic method that combines constraint-based learning approach and maximum likelihood framework (Sella et al., 2017; Affeldt et al., 2016; Verny et al., 2017). MIIC online server (https://miic.curie.fr) aims to learn the most appropriate causal, non-causal or mixed graphical model from the available data (Sella et al., 2017). In brief, starting from a fully connected graph, MIIC iteratively removes dispensable edges, by uncovering significant information contributions from indirect paths. This amounts to progressively uncover the best supported conditional independencies by iteratively ‘taking off’ the most significant indirect contributions of positive conditional 3-point information, from every 2-point (mutual) information.

In order to infer the causal relationship between transcription factors from SCENIC and ferroptosis and EMT markers, we extracted TPM data of malignant tumor cells for further analysis. We used huge. npn function of the huge R package to implement the Gaussianization to help relax the assumption of normality (Zhao et al., 2012). We prepared our dataset formatted as a table with variable names (gene names) specified as column names. Category order data was uploaded with a fourth column named “group” added to regroup. When running MIIC on the web interface, we set the following parameters:

Neff: It is Effective number of independent samples, which is set to −1.

Seed: Value used to initialize the pseudorandom number generator in the random sampling phase which is performed only if an effective number of samples is set.

Complexity: Complexity criterion to take into account finite size effects from N or Neff samples for the network reconstruction. It is set to NML.

Orientation: It is set to yes, which means the orientation of v-structures should be enabled.

Propagation: It is set to yes, which means orientations should be propagated downstream of v-structure.

Latent: It is set to no, which means it is not allowed to detect the effects of unobserved (latent) com-mon causes on the relationships between observed variables, represented by bidirected edges.

### Gene ontology (GO) Analysis and Single-Sample Gene set Enrichment Analysis (ssGSEA)

Differentially expressed genes (DEGs) of subgroups were recognized by the Findmarker function in Seurat. ClusterProfiler (version 4.0.0) was applied to analyze differences between two groups (Wu et al., 2021b). Based on the GO database, functional enrichment analysis of marker genes was performed on each cell to explore their potential biological functions. ssGSEA was applied to calculate the biological function scores of each cell using the R package GSVA (1.38.2) with the “ssgsea” option for the method argument (Hänzelmann et al., 2013). According to the median scores of EMT and Ferroptosis, we divided all ECs into four groups: Ferro_High_EMT_High, Ferro_High_EMT_Low, Ferro_Low_EMT_High, Ferro_Low_EMT_Low.

### Cell–Cell Communication Analysis With iTALK

We mapped the receptor-ligand pairs using iTALK (version 0.1.0) between cell subsets we identified and immune cells to identify cell–cell communications (Wang et al., 2019b). iTALK annotates ligand-receptors into 4 categories: cytokines, growth factors, immune checkpoints and others. For this analysis, we applied two filtering steps of cells inclusion criteria (Chen et al., 2021): Excluded normal lung cells derived from nLung and included all malignant ECs derived from tumors tissues (Puram et al., 2017), Included immune cells identified in the original article: “NK,” “Cytotoxic CD8^+^ T,” “Exhausted CD8^+^ T,” “Treg,” “CD4+ Th.” Considering the better graphics of the chord diagram, we choose to display the top 25 receptor-ligand pairs.

### Deconvolution of scRNA-Seq Data and Correlation to Public Datasets

We extracted the expression matrices of the four groups of cells from the scRNA-seq data. In order to use the cell types in our scRNA-seq to generate the CIBERSORTx feature matrices, we ran the “Create Signature Matrix” module (https://cibersortx.stanford.edu/runcibersortx.php) (Newman et al., 2019). We used the generated feature matrix to perform CIBERSORTx deconvolution on the bulk RNA-seq cohorts. Among the bulk RNA-seq cohorts, TCGA_LUAD was downloaded from GDC Data Portal (https://portal.gdc.cancer.gov/projects/TCGA-LUAD) to observe the overall survival (OS) and TNM stage, while GSE126044 and GSE165252 were included to observe the therapeutic effect of immunotherapy (Cho et al., 2020; van den Ende et al., 2021).

## Data Availability

The original contributions presented in the study are included in the article/Supplementary Material, further inquiries can be directed to the corresponding authors.

## Supplementary Material

Supplementary Figure S1 Some meaningful regulatory relationships. **(A)** undirected network graph visualized using Cytoscape. **(B–D)** The cause-effect relationships between some genes. **(B)** SCP2 promoted PRKAA1, PRKAA2 and CDH2, while inhibiting GPX4 and CAV1. **(C)** HOXB5 promoted CDH2 and PRKAA1, PRKAA2, and is meanwhile promoted by HIF1A, KEAP1 and ATG5. **(D)** CAV1 promoted the expression of GPX4 and CDH1, and inhibits CDH2. **(E,F)** Kaplan-Meier survival curves of GPX4 and PRKAA2.

Supplementary Figure S2
**(A)** The correlation of ssGSEA sore of the malignant epithelial cells. **(B)** The scores of each cell type in the TCGA LUAD cohort. “Ferro_Low_EMT_Low” group (double-low group) had no significant difference in OS and “Ferro_High_EMT_Low” group suggested a good prognosis. **(C,D)** The double-high group had a higher enrichment score in patients with metastases. **(C)** Patients with N1 & N2 stages have significantly higher enrichment scores than those with N0 stage. **(D)** Patients in stage M1 received significantly higher scores than those in stage M0.

Supplementary Figure S3 Analysis of molecular correlations in TCGA dataset.
