# Supplementary figures and images for: Single-Cell RNA-Seq Reveals the Promoting Role of Ferroptosis Tendency During Lung Adenocarcinoma EMT Progression
Source: Front Cell Dev Biol. 2022 Jan 20;9:822315. doi: 10.3389/fcell.2021.822315 (PMC8810644; doi:10.3389/fcell.2021.822315)

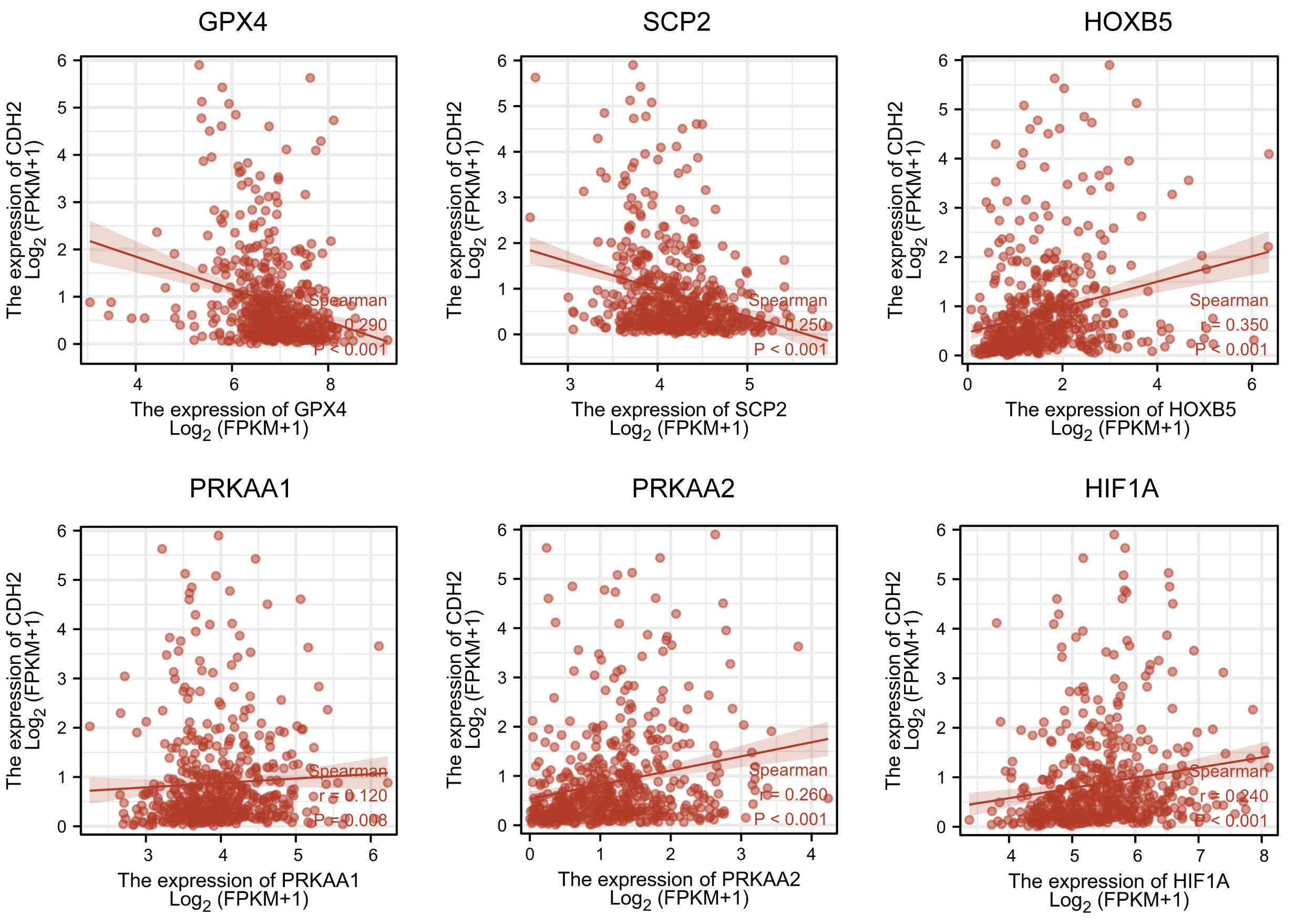

Supplement: Supplementary file 3 [file Image3.TIF]

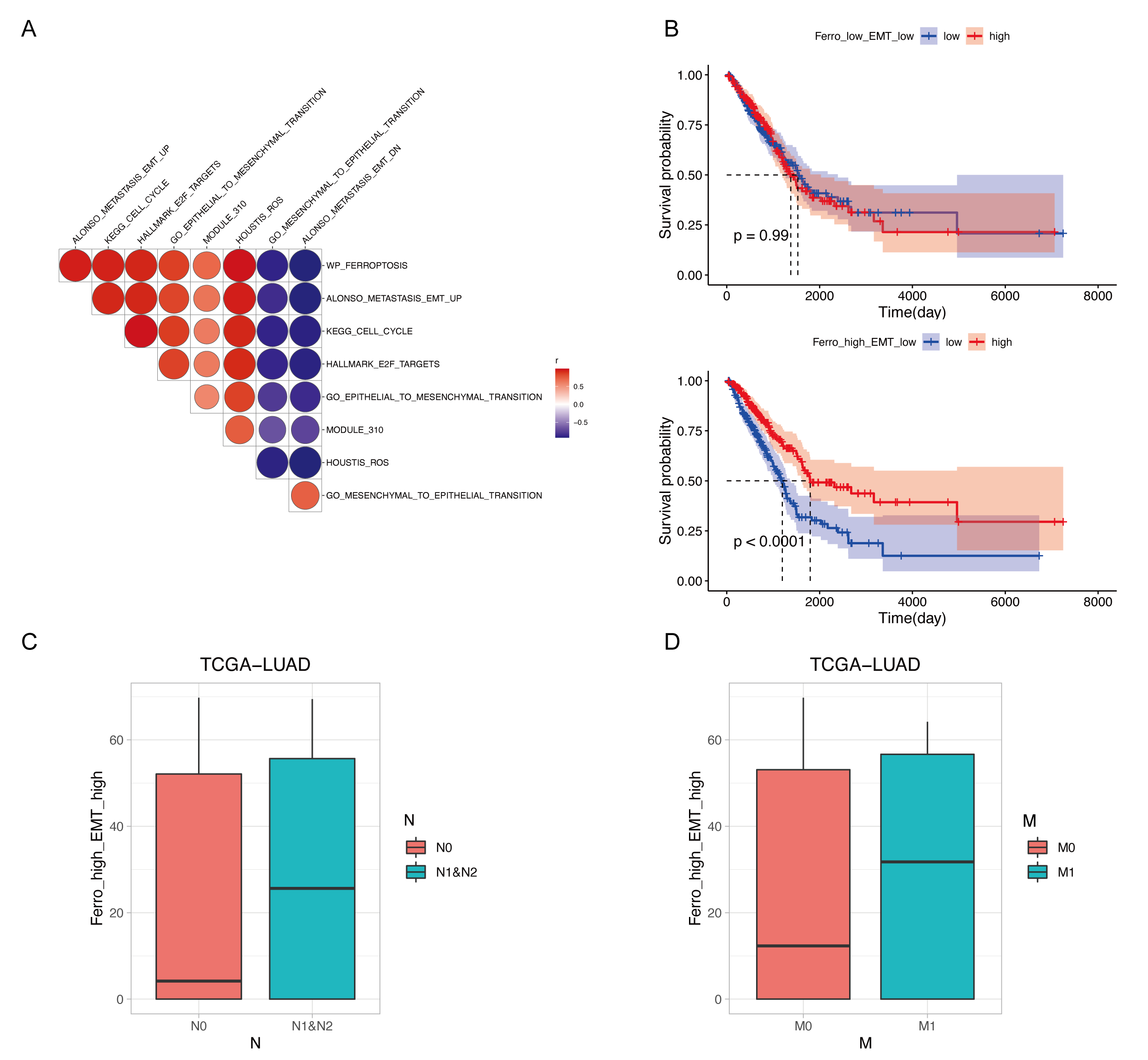

Supplement: Supplementary file 4 [file Image2.TIF]

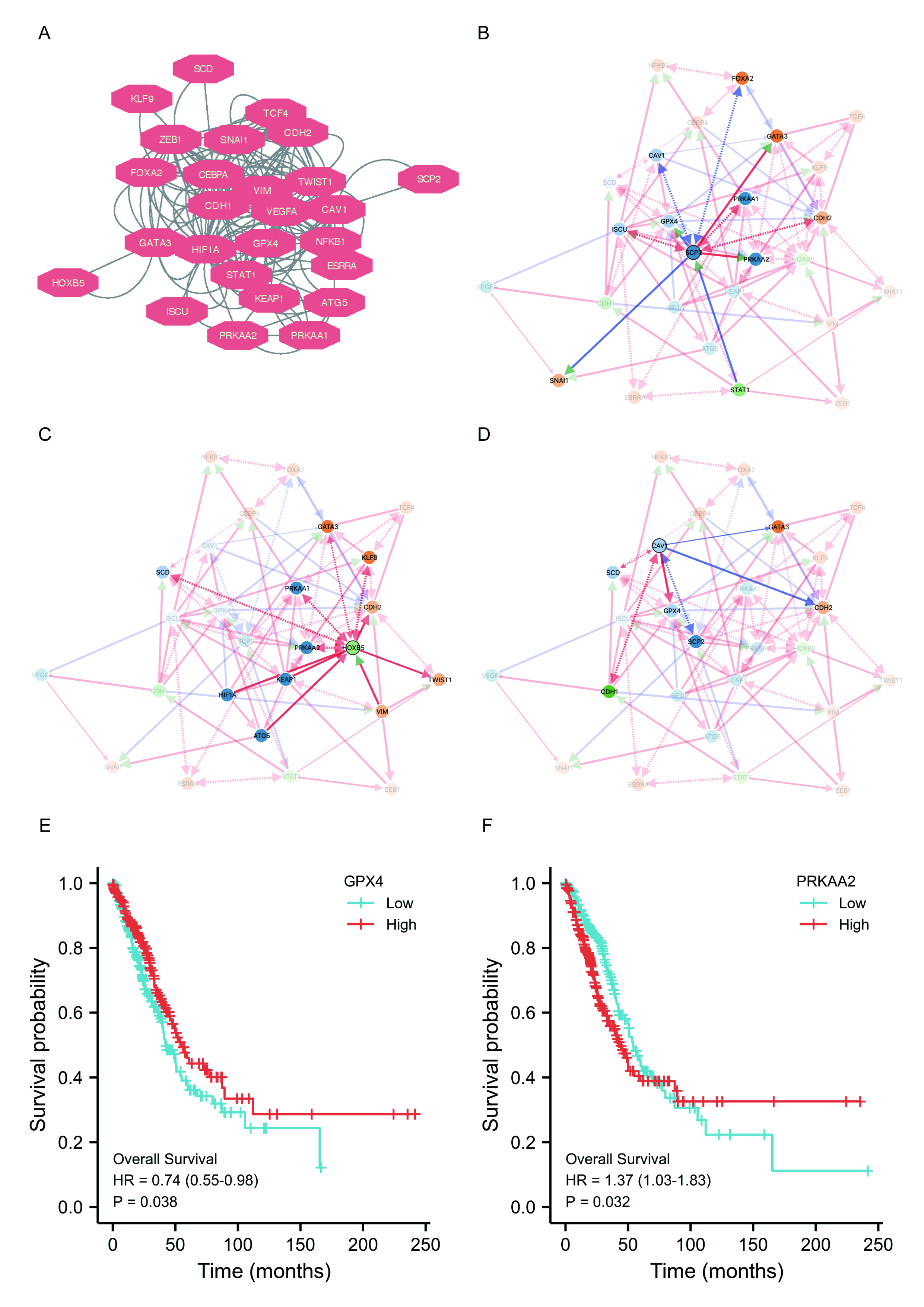

Supplement: Supplementary file 5 [file Image1.TIF]
